# Evaluation of techniques to improve a deep learning algorithm for the automatic detection of intracranial haemorrhage on CT head imaging

**DOI:** 10.1186/s41747-023-00330-3

**Published:** 2023-04-10

**Authors:** Melissa Yeo, Bahman Tahayori, Hong Kuan Kok, Julian Maingard, Numan Kutaiba, Jeremy Russell, Vincent Thijs, Ashu Jhamb, Ronil V. Chandra, Mark Brooks, Christen D. Barras, Hamed Asadi

**Affiliations:** 1 Melbourne Medical School, The University of Melbourne, Melbourne, VIC Australia; 2 Department of Biomedical Engineering, The University of Melbourne, Melbourne, VIC Australia; 3 IBM Research Australia, Melbourne, VIC Australia; 4 Interventional Radiology Service, Department of Radiology, Northern Health, Epping, VIC Australia; 5 School of Medicine, Faculty of Health, Deakin University, Burwood, VIC Australia; 6 Interventional Neuroradiology Unit, Monash Health, Clayton, VIC Australia; 7 Faculty of Medicine Nursing and Health Sciences, Monash University, Clayton, VIC Australia; 8 Department of Radiology, St Vincent’s Hospital, Melbourne, VIC Australia; 9 Department of Radiology, Austin Hospital, Melbourne, VIC Australia; 10 Department of Neurosurgery, Austin Hospital, Melbourne, VIC Australia; 11 Stroke Theme, Florey Institute of Neuroscience and Mental Health, Melbourne, VIC Australia; 12 Department of Neurology, Austin Health, Melbourne, VIC Australia; 13 Interventional Neuroradiology Service, Department of Radiology, Austin Hospital, Melbourne, VIC Australia; 14 South Australian Institute of Health and Medical Research, Adelaide, South Australia Australia; 15 School of Medicine, The University of Adelaide, Adelaide, South Australia Australia

**Keywords:** Artificial intelligence, Deep learning, Intracranial haemorrhages, Radiographic image interpretation (computer-assisted), Tomography (x-ray computed)

## Abstract

**Background:**

Deep learning (DL) algorithms are playing an increasing role in automatic medical image analysis.

**Purpose:**

To evaluate the performance of a DL model for the automatic detection of intracranial haemorrhage and its subtypes on non-contrast CT (NCCT) head studies and to compare the effects of various preprocessing and model design implementations.

**Methods:**

The DL algorithm was trained and externally validated on open-source, multi-centre retrospective data containing radiologist-annotated NCCT head studies. The training dataset was sourced from four research institutions across Canada, the USA and Brazil. The test dataset was sourced from a research centre in India. A convolutional neural network (CNN) was used, with its performance compared against similar models with additional implementations: (1) a recurrent neural network (RNN) attached to the CNN, (2) preprocessed CT image-windowed inputs and (3) preprocessed CT image-concatenated inputs. The area under the receiver operating characteristic curve (AUC-ROC) and microaveraged precision (mAP) score were used to evaluate and compare model performances.

**Results:**

The training and test datasets contained 21,744 and 491 NCCT head studies, respectively, with 8,882 (40.8%) and 205 (41.8%) positive for intracranial haemorrhage. Implementation of preprocessing techniques and the CNN-RNN framework increased mAP from 0.77 to 0.93 and increased AUC-ROC [95% confidence intervals] from 0.854 [0.816–0.889] to 0.966 [0.951–0.980] (*p*-value = 3.91 × 10^−12^).

**Conclusions:**

The deep learning model accurately detected intracranial haemorrhage and improved in performance following specific implementation techniques, demonstrating clinical potential as a decision support tool and an automated system to improve radiologist workflow efficiency.

**Key points:**

• The deep learning model detected intracranial haemorrhages on computed tomography with high accuracy.

• Image preprocessing, such as windowing, plays a large role in improving deep learning model performance.

• Implementations which enable an analysis of interslice dependencies can improve deep learning model performance.

• Visual saliency maps can facilitate explainable artificial intelligence systems.

• Deep learning within a triage system may expedite earlier intracranial haemorrhage detection.

**Supplementary Information:**

The online version contains supplementary material available at 10.1186/s41747-023-00330-3.

## Background

Intracranial haemorrhage is an important neurological emergency characterised by bleeding into the cranial vault. Its subtypes depend on the location of haemorrhage: into the brain parenchyma (intracerebral haemorrhage (ICH)), the subarachnoid space (subarachnoid haemorrhage (SAH)), the ventricles (intraventricular haemorrhage (IVH)), the space between the dura mater and skull (extradural haemorrhage (EDH)) or the space between the dura mater and arachnoid mater (subdural haemorrhage (SDH)). Intracranial haemorrhage has reported 30-day mortality rates of up to 61% and low rates of full functional independence amongst survivors [1].

Delays in the detection of intracranial haemorrhage translate into delays in specialist referral and active management, leading to potentially preventable cerebral injury and morbidity/mortality [2]. The practical gold standard imaging modality is non-contrast computed tomography (NCCT) of the head. However, due to the rising complexity and volume of contemporary imaging studies, the identification of positive head NCCT studies may be delayed by competing acute imaging studies. Additionally, after-hours or rural settings may lack experienced clinicians/radiologists, compounding the challenges of prompt, accurate haemorrhage detection. Significant discrepancies in image interpretation have been reported between experienced radiologists and junior radiologists/emergency physicians [3–5], with missed SDHs, SAHs [3], fractures and chronic ischaemic foci [4]. Hence, an automated process has the potential to reduce these misdiagnoses and expedite the evaluation and management of intracranial haemorrhages.

In recent years, deep learning (DL) algorithms have been proposed for automatic medical image analysis. DL is based on an artificial neural network structure inspired by the human brain. In image analysis, DL networks such as convolutional neural networks (CNNs) learn hierarchical feature representations from images, automatically building high-level information from low-level features. This enables sensitivity to minute image details while retaining insensitivity to large irrelevant variations in the background [6]. Commercial solutions exist for haemorrhage detection (*e.g.,* RAPID ICH, iSchemaView, Inc. and Viz ICH, Viz.ai, Inc.), However, their non-open-source nature limits disclosure of the DL architectures and implementation techniques used. This subsequently reduces public insight into how these algorithms may be improved and prevents public benchmarking to evaluate their performances transparently and robustly.

This study aims to implement a DL model for the automatic detection of intracranial haemorrhage and subtypes on NCCT head studies. However, the analysis of volumetric scans presents a unique challenge, as most computer vision tasks focus on two-dimensional (2D) image analysis. Several studies addressed this via the use of a three-dimensional (3D) CNN model [7–9] but due to the “curse of dimensionality”, each increase in input data dimensionality exponentially increases the amount of data required to train a model. An alternative to this is a joint 2D CNN-recurrent neural network (RNN) model, which combines the image analysis capabilities of a CNN with an RNN ability to analyse sequences, thus capturing the relationship between all slices in a volume [10]. Other studies used slice combination methods, feeding preprocessed image data containing consecutive slices—slices immediately superior and inferior to each input slice—into the DL model [11, 12]. We aimed to extend these approaches by combining both techniques.

Additionally, although previous studies have demonstrated promising performance with CNN-RNN architectures, their validity is limited by the testing methodology used. Ye et al. [10] and Grewal et al. [13] used split-sample validation, with the training and test samples derived from the same dataset. This is a less robust method of validation compared to testing on an independent dataset acquired from a different location with different scanners/scanning protocols.

Thus, the objectives of this study are threefold: (1) to design a DL model for the detection, subtyping and localisation of intracranial haemorrhages on NCCTs; (2) to compare several implementation techniques and evaluate their benefit; and (3) to validate the performance of the model on an independent retrospective dataset.

## Methods

The DL model implemented in this study was programmed in the Python programming environment (Python Software Foundation, https://www.python.org/). It was trained and tested on open-source datasets containing radiologist-labelled NCCT head studies. To enhance model performance, raw input scans were preprocessed using image windowing and slice concatenation techniques. This preprocessed data was subsequently analysed by a joint CNN-RNN DL model. Finally, in addition to producing predictions, the model generated output saliency heatmap images, with the aim of increasing the explainability of the algorithm. The following subsections outline these processes in detail.

### Datasets

Two open-source retrospective datasets were used in this study. Both datasets were composed of de-identified data, licensed for non-commercial and academic use. The study was approved by the local institutional ethics review board.

The first dataset, the Kaggle dataset, was obtained from a 2019 online Kaggle challenge hosted by the Radiological Society of North America [14]. This contained 752,803 NCCT slices (21,744 studies), collected across four research institutions (Stanford University, Thomas Jefferson University, Unity Health Toronto and Universidade Federal de São Paulo). The data had been manually labelled by sixty radiologists from the American Society of Neuroradiology. Each scan was annotated at the slice level, labelled with the presence or absence of the following six classes: EDH, ICH, IVH, SAH, SDH, and intracranial haemorrhage (*i.e.,* any haemorrhage subtype). Each scan may have more than one haemorrhage subtype.

The second dataset, the CQ500 dataset from qure.ai, was previously used in a study by Chilamkurthy et al. [15], collected from the Centre for Advanced Research in Imaging, Neurosciences and Genomics, in New Delhi, India [16]. This dataset contained 193,317 slices (491 studies) and excluded postoperative scans and scans of patients younger than 7 years. The data included annotations, manually labelled by three radiologists with experience of 8, 12, and 20 years respectively in cranial NCCT interpretation. Each scan was annotated at the subject level by each radiologist, with class labels matching those of the Kaggle dataset. The majority vote of these three radiologists’ annotations was used as the gold standard. Inter-rater reliability between radiologists was highest for intracranial haemorrhage and ICH (Fleiss *κ* = 0.78 for both) and lowest for SDH (Fleiss *κ* = 0.54), as detailed in Supplementary Table S1.

Both datasets encompassed data collected from institutions across separate geographic locations (the USA, Canada, Brazil, India), using different computed tomography (CT) scanners and protocols. The characteristics of the CT studies acquired in both datasets are detailed in Supplementary Table S2. CT studies in both datasets contained varying numbers of slices (12–548) and varying slice thicknesses (0.625–7 mm). Most CT studies had a slice thickness of 5 mm. More accurate patient demographics were unable to be assessed, as this information was not provided by the publishers of the open-source datasets.

The Kaggle dataset was used to develop and train the model. The CQ500 dataset was used as an independent dataset for testing and verifying the performance of the trained model. Testing was carried out at the subject level instead of the slice level. A previous study which used the CQ500 dataset also used it as a test dataset [15]; however, their DL model approach and training data differed from the present study.

### Data preprocessing

To improve the performance of the DL model, all CT images were preprocessed prior to being fed into the model. Two separate preprocessing pipelines were used.

In the first pipeline, an image windowing technique was used to mimic the clinical workflow of radiologists (Supplementary Fig. S1). This involves adjusting the window width (WW) and window level (WL) display settings of the CT image, to accentuate particular tissues or abnormalities being evaluated. Although several studies have incorporated this into the preprocessing steps of their DL implementations [8, 10, 13, 15, 17], the benefits of windowing have not previously been reported. Hence, we sought to clarify the extent of the impact of adding such a step. To incorporate this, each one-channel DICOM CT slice was converted into a three-channel 8-bit JPEG image (similar to the three-channel RGB format), with each channel set to a specific window level (WL) and window width (WW) setting: brain window (WL = 40, WW = 80), subdural window (WL = 80, WW = 200) and soft tissue window (WL = 40, WW = 380).

In the second pipeline, a slice concatenation technique was used to mimic the way in which radiologists integrate information from adjacent slices when interpreting volumetric scans (Supplementary Fig. S2). Each one-channel DICOM CT slice was converted into a three-channel JPEG image, with each channel corresponding to the current slice and the slices immediately superior and inferior to it. These slices were brain-windowed. In cases with unavailable adjacent slice(s), the current slice was used instead.

In both pipelines, all image slices were then downsampled from 512 × 512 to 480 × 480 pixels to reduce memory usage. In addition, to synthetically “generate” more data for the model to train on and to improve the generalisability of the model, data augmentation was also performed. This involved geometrical transformations, with random extents of rotation (± 0–20°), scaling (by a factor of ± 0–0.05), shifting of height and width (by a factor of ± 0–0.05) and horizontal flipping applied to the images.

### Deep learning model workflow

Two types of CNN models were trained: one using the image-windowed preprocessing pipeline (CNN_wdw_) and another using the slice-concatenated preprocessing pipeline (CNN_slc_). Both models were trained to detect the presence of any of the following predetermined six types of intracranial haemorrhage. A detected haemorrhage in any slice indicated positivity for haemorrhage for the patient. These two models were then combined into an ensemble (CNN_ens_). Predictions from CNN_ens_ were based on the unweighted average of the probabilities predicted by both CNN_wdw_ and CNN_slc_. The final joint CNN-RNN model (CNN_ens_-RNN) was created by joining the outputs of CNN_ens_ to an RNN.

An important issue plaguing DL models is their lack of interpretability. To address this, we implemented a technique of providing “visual explanations” for the model predictions. This outputs a heatmap, which highlights the CT image pixels that contribute most significantly to the model prediction. This served the purpose of (1) increasing the explainability of the model and (2) indicating the region of haemorrhage(s). The heatmap was generated by applying the Gradient-weighted Class Activation Mapping (Grad-CAM) technique [18] on CNN_wdw_. CNN_wdw_ was selected for this purpose as Grad-CAM cannot be applied to ensemble models, and CNN_wdw_ takes more varied pixel information from a single CT slice compared to CNN_slc_.

### Deep learning model training procedures

The training Kaggle dataset was randomly split into training (80%) and validation (20%) sets. The training set was used to fit the model parameters. The validation set was used to further tune hyperparameters, to prevent the model from overfitting to the training set.

The DL model was implemented in the Python programming language (Python 3.6) using the PyTorch DL framework (version 1.4.0). The CNN used was an ImageNet-pretrained model with a ResNeXt architecture and received as input 2D images of 480 × 480 pixel size [19]. We used the Adam optimiser and weighted binary cross-entropy loss as the loss function. A constant learning rate of 5 × 10^−5^ was used. When combined end-to-end, the CNN-RNN was composed of the same CNN model, with an additional RNN containing two bidirectional long short-term memory layers. The CNN outputs (obtained from the layer immediately before the final fully connected layer) were used as input into the RNN. The model is illustrated in Supplementary Fig. S3.

Training was performed on a high-performance computing system with two Intel Xeon E5-2650 central processing unit processors (24 cores), four NVIDIA P100 graphics processing units and 128 GB of random-access memory. Each model took about 50 h to train.

### Statistical analysis

All statistical analyses were performed using the Python modules scikit-learn (version 0.22.1) and statsmodels (version 0.12.2). The performance of the DL model on the test dataset was evaluated using the following metrics: accuracy, sensitivity, specificity, positive likelihood ratio, negative likelihood ratio, area under the receiver operating characteristic curve (AUC-ROC), area under the precision-recall curve (AUC-PR) and microaveraged precision score (mAP). Ninety-five per cent confidence intervals for accuracy, sensitivity and specificity were calculated using the exact Clopper-Pearson method based on *β* distribution [20].

For each class, receiver operating characteristic (ROC) curves [21] were obtained by plotting the true positive rate (sensitivity) against the false positive rate (1 − specificity) at various discriminative threshold settings. Two operating points were selected—a “high sensitivity” and a “balanced” operating point—based on performance on the validation dataset. A “high sensitivity” operating point was chosen on the ROC curve, which maximised sensitivity while ensuring a minimum specificity of 80%. If the sensitivity value at this point was less than 93%, another point was chosen with a minimum specificity of 70%. The decision to select an operating point that placed greater emphasis on sensitivity, over specificity, was based on the DL model aim to be used as a triage tool. A second more “balanced” operating point was also chosen, which maximised the Youden index [22] while ensuring a minimum sensitivity of 85%.

Precision-recall (PR) curves were obtained by plotting precision against recall at various discriminative threshold settings. Compared to ROC curves, PR curves are more reliable in datasets containing class imbalance [23–25], such as in this application where certain haemorrhage subtypes are more common. The mAP, a single informative metric that summarises PR curves across all classes, as well as the AUC-ROC (for the detection of the class ‘any intracranial haemorrhage’), were used to compare specific model implementations. The DeLong test was used to evaluate the statistical significance between AUC-ROCs. The McNemar test was used to assess if the cases of false negative and false positives were significantly different between models.

## Results

### Datasets

The DL models were trained on the Kaggle dataset and tested on the CQ500 dataset. Both datasets were intrinsically imbalanced (Table 1). EDH-positive scans made up a substantially smaller proportion of the data in both datasets (1.6% and 2.6% of each dataset), as compared to the other haemorrhage subtypes (17.0–40.8% and 5.7–41.8%). Both datasets contained predominantly images negative for haemorrhage (59.2% and 58.2% of each dataset).

**Table 1:** Proportions of each class label in the Kaggle and CQ500 datasets

	**Training (Kaggle dataset)**	**Test (CQ500 dataset)**
No haemorrhage	12,862 (59.2%)	286 (58.2%)
Intracranial haemorrhage	8,882 (40.8%)	205 (41.8%)
EDH	354 (1.6%)	13 (2.6%)
ICH	5,321 (24.5%)	134 (27.3%)
IVH	3,692 (17.0%)	28 (5.7%)
SAH	3,932 (18.1%)	60 (12.2%)
SDH	3,812 (17.5%)	53 (10.8%)

Data is provided in *n* volumetric scans (%)

*EDH* Extradural haemorrhage, *ICH* Intracerebral haemorrhage, *IVH* Intraventricular haemorrhage, *SAH* Subarachnoid haemorrhage, *SDH* Subdural haemorrhage

### Comparison of model performance

**Fig. 1:**
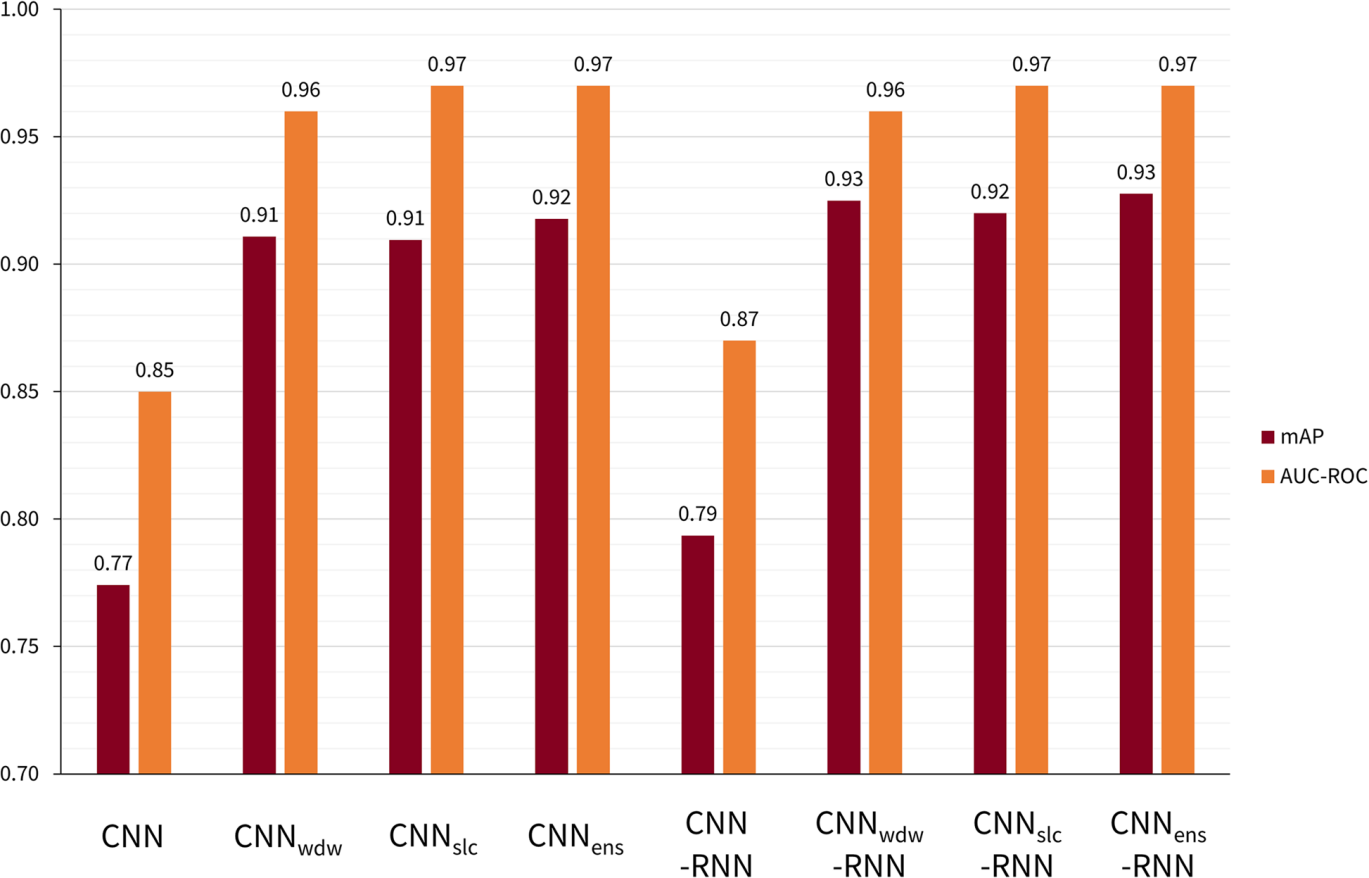
Comparison of model performances with different preprocessing and addition of an RNN. CNN denotes the model composed of a CNN, with no preprocessing image techniques or RNN added. CNN_wdw_ denotes the CNN model trained only with the image windowing preprocessing pipeline. CNN_slc_ denotes the CNN model trained only with the adjacent slice concatenation preprocessing pipeline. CNN_ens_ denotes the ensemble model combining CNN_wdw_ with CNN_slc_. CNN_wdw_-RNN denotes the CNN_wdw_ model joined to an RNN. CNN_slc_-RNN denotes the CNN_slc_ model joined to an RNN. CNN_ens_-RNN denotes the CNN_ens_ model joined to an RNN. *AUC-ROC* Area under the receiver operating characteristic curve, *CNN* Convolutional neural network, *mAP* Average precision score (microaveraged across all six haemorrhage classes), *RNN* Recurrent neural network

The impact of the techniques used to enhance DL model performance was evaluated using mAP and AUC-ROC scores (Fig. 1).

For the detection of any intracranial haemorrhage subtype, the addition of both the image windowing and slice concatenation preprocessing pipelines, along with the RNN attached to the CNN, increased mAP from 0.77 to 0.93 and increased AUC-ROC from 0.85 to 0.97 (DeLong *p*-value = 3.91 × 10^−12^).

Compared to no preprocessing (CNN), the use of the image windowing preprocessing pipeline (CNN_wdw_) increased mAP from 0.77 to 0.91 and increased AUC-ROC from 0.85 to 0.96 (DeLong *p*-value = 5.10 × 10^−12^). Compared to no preprocessing (CNN), the use of the slice concatenation preprocessing pipeline (CNN_slc_) increased mAP from 0.77 to 0.91 and increased AUC-ROC from 0.85 to 0.97 (DeLong *p*-value = 1.90 × 10^−12^). Combining both pipelines (CNN_ens_) resulted in a mAP of 0.92 and an AUC-ROC of 0.97, which was not statistically significant when compared to using only the image windowing pipeline (CNN_wdw_) or only the slice concatenation pipeline (CNN_slc_) (DeLong *p*-values of 0.065 and 0.823 respectively). The McNemar test was significant for the former but not the latter (McNemar *p*-values of 0.014 and 0.052, respectively).

The addition of the RNN to the CNN increased mAP from 0.77 to 0.79 and increased AUC-ROC from 0.85 to 0.87 (DeLong *p*-value = 0.017). The McNemar test was not significant (*p* = 1.000). Compared to without the RNN, the addition of the RNN with either the image windowing pipeline, or the slice concatenation pipeline, or both pipelines led to increases in mAP: 0.91 to 0.93 for CNN_wdw_ *versus* CNN_wdw_-RNN, 0.91 to 0.92 for CNN_slc_ *versus* CNN_slc_-RNN, and 0.92 to 0.93 for CNN_ens_ *versus* CNN_ens_-RNN. The changes in AUC-ROC were not statistically significant (DeLong *p*-values of 0.922, 0.902, and 0.750 respectively); however, the McNemar test was highly significant (*p* = 1.39 × 10^−17^, 5.23 × 10^−30^, 2.25 × 10^−24^).

Further data comparing the performance of each of these models can be found in Supplementary Tables S3 and S4.

### Model performance on haemorrhage detection

**Fig. 2:**
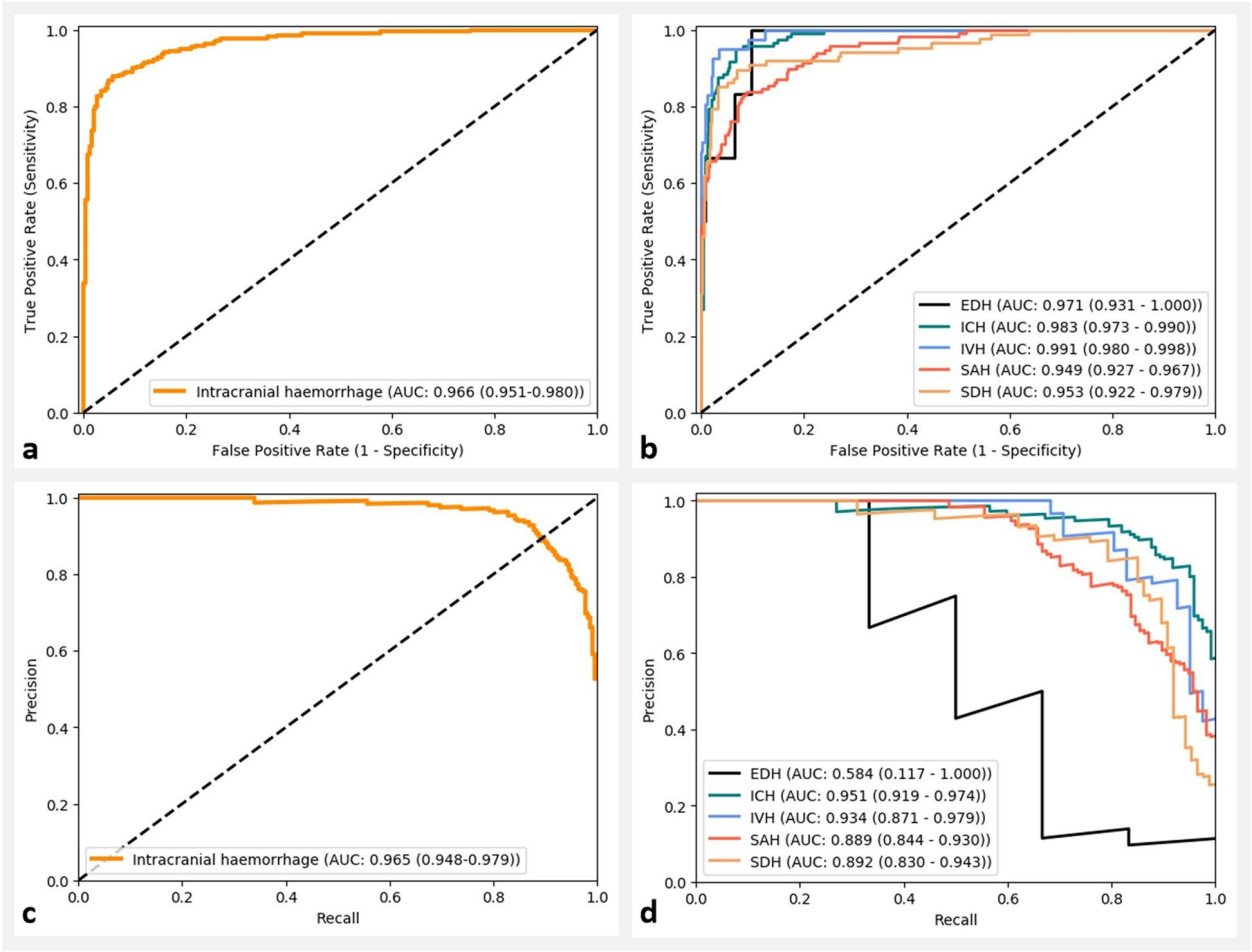
ROC and PR curves for the deep learning model on the CQ500 dataset. The top two graphs show the ROC curves (**a** intracranial haemorrhage; **b** each haemorrhage subtype). The bottom two graphs show the PR curves (**c** intracranial haemorrhage; **d** each haemorrhage subtype). Ninety-five per cent confidence intervals are provided in parentheses for each AUC. *AUC* Area under the curve, *EDH* Extradural haemorrhage, *ICH* Intracerebral haemorrhage, *IVH* Intraventicular haemorrhage, *PR* Precision-recall, *ROC* Receiver operating characteristic, *SAH* Subarachnoid haemorrhage, *SDH* Subdural  haemorrhage

Figure 2 and Table 2 summarise the performance of the final CNN_ens_-RNN model on the CQ500 dataset. The model achieved AUC-ROCs of 0.966, 0.971, 0.983, 0.991, 0.949, and 0.953 and AUC-PRs of 0.965, 0.584, 0.951, 0.934, 0.889, and 0.892 for the detection of intracranial haemorrhage, EDH, ICH, IVH, SAH, and SDH, respectively. At the high-sensitivity operating point (sensitivities from 0.95 to 1.00), the accuracy range was 0.77–0.90, with specificities from 0.73 to 0.90. At the balanced operating point, the accuracy range was 0.86–0.96, with sensitivities from 0.87 to 1.00 and specificities from 0.85 to 0.96.

**Table 2:** DL model performance on the CQ500 test dataset at two selected operating points

	**Accuracy**	**Sensitivity**	**Specificity**	**Positive likelihood ratio**	**Negative likelihood ratio**
High-sensitivity operating point					
Intracranial haemorrhage	0.88 (0.84–0.90)	0.95 (0.91–0.97)	0.81 (0.76–0.86)	5.100	0.061
EDH	0.90 (0.87–0.93)	1.00 (0.54–1.00)	0.90 (0.87–0.93)	10.173	0.000
ICH	0.87 (0.83–0.90)	0.99 (0.96–1.00)	0.83 (0.78–0.86)	5.700	0.009
IVH	0.89 (0.85–0.91)	1.00 (0.91–1.00)	0.88 (0.84–0.91)	8.051	0.000
SAH	0.79 (0.75–0.82)	0.96 (0.90–0.99)	0.74 (0.69–0.78)	3.622	0.058
SDH	0.77 (0.73–0.81)	0.94 (0.87–0.98)	0.73 (0.68–0.77)	3.497	0.078
Balanced operating point					
Intracranial haemorrhage	0.91 (0.88–0.94)	0.88 (0.83–0.92)	0.94 (0.91–0.97)	15.400	0.129
EDH	0.90 (0.87–0.93)	1.00 (0.54–1.00)	0.90 (0.87–0.93)	10.173	0.000
ICH	0.94 (0.91–0.96)	0.95 (0.90–0.98)	0.93 (0.90–0.96)	14.341	0.053
IVH	0.96 (0.94–0.98)	0.95 (0.83–0.99)	0.96 (0.94–0.98)	26.349	0.051
SAH	0.86 (0.82–0.89)	0.87 (0.80–0.93)	0.85 (0.81–0.89)	5.927	0.150
SDH	0.93 (0.90–0.95)	0.90 (0.81–0.95)	0.93 (0.90–0.95)	13.185	0.111

95% CIs are provided in parentheses for accuracy, sensitivity and specificity

The greater the positive likelihood ratio, the greater the effect on the post-test probability of disease given a positive test result (0–5: slight increase, 5–10: moderate increase, > 10: large increase)

The smaller the negative likelihood ratio, the greater the effect on the post-test probability of disease given a negative test result (0.5–1: slight decrease, 0.2–0.5: moderate decrease, 0–0.1: large decrease)

*DL* Deep learning, *EDH* Extradural haemorrhage, *ICH* Intracerebral haemorrhage, *IVH* Intraventricular haemorrhage, *SAH* Subarachnoid haemorrhage, *SDH* Subdural haemorrhage

The time taken for each model to perform inference on the CQ500 dataset was about 1 h. The slowest model spent 1 h and 6 min, such that the average time taken to analyse a single slice was 0.0205 s. Given an average CT head scan contains about 30 axial images, this would take approximately 0.615 s to analyse.

### Model visualisation

**Fig. 3:**
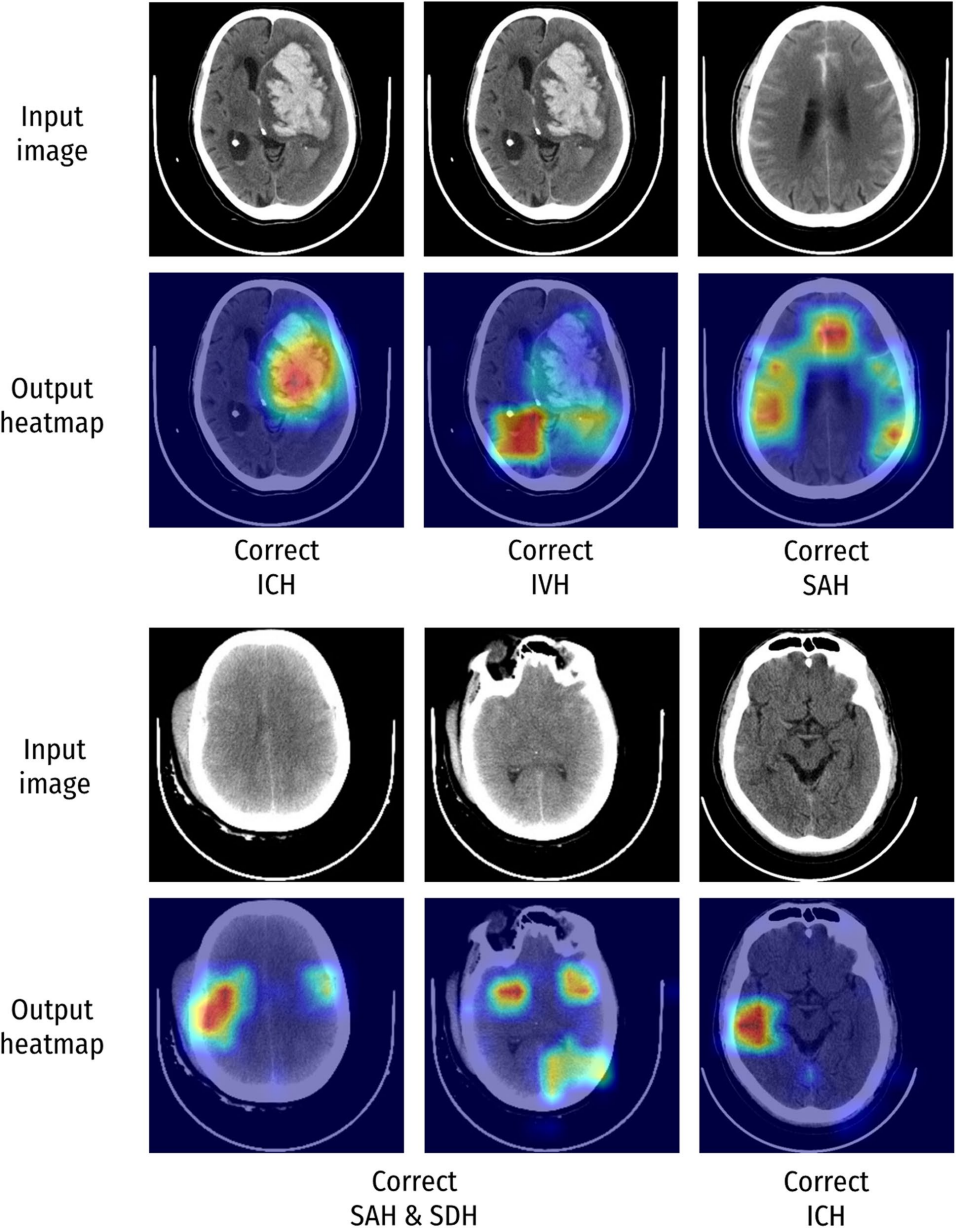
Visualisation of correct model predictions using heatmap images. Examples of the regions that contributed to the model decision in predicting the presence of haemorrhages. The warmer the colour (red > orange > yellow > green > blue), the greater the contribution of the image pixel to the prediction. *EDH* Extradural haemorrhage, *ICH* Intracerebral haemorrhage, *IVH* Intraventricular haemorrhage, *SDH* Subdural haemorrhage

**Fig. 4:**
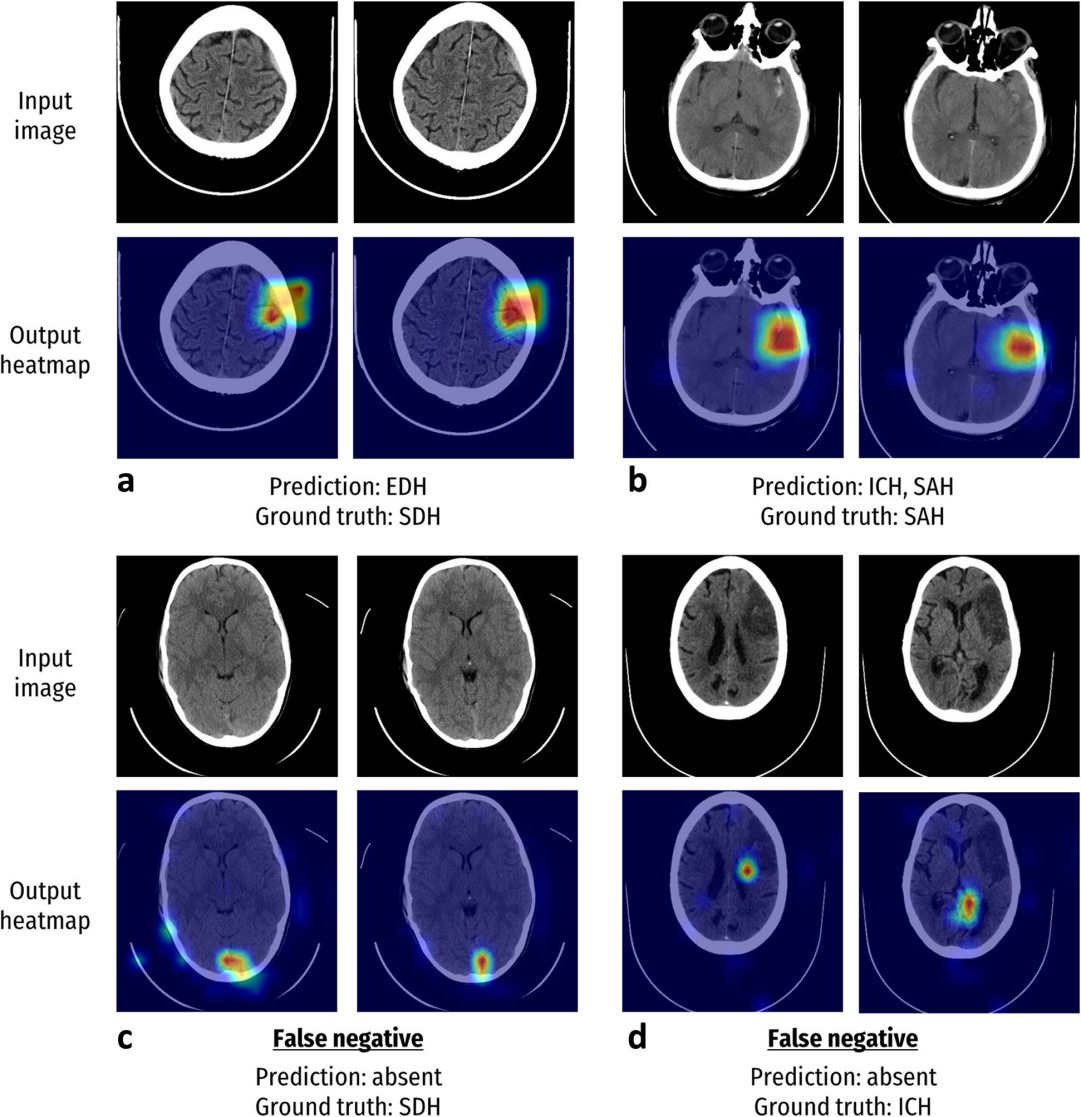
Visualisation of incorrect model predictions using heatmap images. Examples of the regions which contributed to the model decision in predicting haemorrhages. The warmer the colour (red > orange > yellow > green > blue), the greater the contribution of the image pixel to the prediction. **a** The model predicted an EDH, whereas the ground truth consensus label determined by radiologists was an SDH. Notably, the haemorrhage had a biconvex shape more closely associated with EDHs. **b** The model predicted ICH and SAH. Although one radiologist identified both SAH and ICH, the ground truth consensus only indicated the presence of a SAH. However, there are several subtle areas indicating possible ICH. **c** False negative in which the model missed a SDH. Although the model detected a suspicious area as indicated on the heatmap, it did not reach a sufficient threshold to be classified as a haemorrhage. Stagnant blood in the dural sinuses, which are benign and more common, can appear similar to SDH in this area, which may have contributed to the model error. **d** False negative in which the model missed an ICH. The heatmap indicated that the model did not appropriately detect areas of abnormality. The presence of chronic infarction here may have affected the model performance. *EDH* Extradural haemorrhage, *ICH* Intracerebral haemorrhage, *IVH* Intraventricular haemorrhage, *SDH* Subdural haemorrhage

In addition to producing a prediction of the presence or absence of haemorrhage, our implementation also generated saliency heatmaps, highlighting the input image pixels which contributed most significantly to the final model prediction. These heatmaps were qualitatively assessed. Figures 3 and 4 depict examples of these images when the DL model (CNN_wdw_) was applied to the CQ500 dataset. The heatmaps in Fig. 3 indicate that the model largely based its predictions on haemorrhagic areas and, depending on the haemorrhage subtype, focused on different areas. In situations where the model made false predictions (Fig. 4), the heatmap can help to identify the image pixels that the model had misinterpreted, thus providing insights into these incorrect predictions, as demonstrated in the bottom two image sets. The top two image sets of Fig. 4 indicate further discrepancies between the model prediction and the ground truth radiologist consensus. However, in these cases, there was also disagreement amongst the radiologists. Such contentious cases demonstrate the difficulty of assessing the true performance of the model, especially where there is a lack of an objective ground truth.

## Discussion

In this study, we developed a highly accurate DL model for the automatic detection of intracranial haemorrhage on NCCT studies. Our implementation additionally subtypes the haemorrhage and produces Grad-CAM-generated heatmaps, which increases the explainability of the algorithm through visual interpretation.

Our model performance was validated on an independent retrospective test set, previously used by Chilamkurthy et al. [15]. Despite utilising a smaller dataset of images for training compared to the aforementioned authors, our model achieved superior performance. Additionally, the performance of our model on intracranial haemorrhage subtype classification was comparable to or better than other studies currently in the literature [7–12, 15]. It achieved a superior AUC-ROC over previously reported 3D CNN implementations [7, 8]. Notably, although Chang et al. [26] and Kuo et al. [11] demonstrated results exceeding or comparable to ours respectively, the architecture of their algorithms (mask R-CNN and patch-based fully convolutional network respectively) necessitated pixel-level annotations. Such annotations are time-consuming to obtain and less scalable with time as datasets grow. Furthermore, previous studies have used split-sample validation to verify their algorithms’ performance [7, 9, 10, 27–29]. Thus, although comparisons of performances were similar in some cases, our results were able to demonstrate greater validity through testing on an independent dataset.

The performance of our model may be attributed to several design implementations. Firstly, we leveraged transfer learning: instead of initialising a model with random weights, we used a model pretrained on the large ImageNet dataset [30], fine-tuning it for the current application. Transfer learning is known to reduce the amount of data required to train a model [31], and two studies have shown the advantages of using a pretrained model over a model trained from scratch for this application [27, 28]. However, these studies did not validate their model on an independent test dataset, limiting reliability. Secondly, we used image windowing, a technique frequently used by radiologists to accentuate specific regions of interest. Although other studies in the literature also implemented this, detailed quantification of the improvement in performance with the addition of this technique has not been reported [8, 10, 12, 13, 15, 17]. Our study addressed this, providing important evidence for the role of image preprocessing in improving the quality of data fed to DL models and its subsequent effects on performance. Thirdly, our implementation was able to incorporate the full spatial information of all slices within a CT volume. We added and compared several implementations to our 2D CNN model that enabled the analysis of interslice dependencies. We (1) concatenated adjacent slices together during the image preprocessing step and (2) used an RNN to analyse sequential slice information. Other studies have shown the benefit of each of these techniques [10–12]; however, none has integrated both into a single model. Although we did not find a statistically significant improvement in performance when combining both methods, McNemar tests did indicate that the disagreement between the models was significant.

Another notable feature of this study is the use of purely open-source datasets, sourced from multiple institutions. These aggregated datasets were collected using multiple different CT scanners from different manufacturers, with various acquisition protocols. Additionally, the patient population was more diverse than data sourced from a single institution or geographic location. By training and testing our model on such data, it ensures generalisability to other CT data and mitigates performance errors caused by factors related to CT scan acquisition. Although previous studies have shown comparable results, they were limited in validity due to the use of split-sample validation.

Furthermore, this study highlights the importance of such open-source datasets in supporting continuing research in this area. Similar to the ImageNet challenge [32], datasets such as these can be used as a “benchmarking” challenge, allowing the performance of different models to be more reliably compared via validation on the same dataset.

This study also sought to address an important limitation of DL models. DL models are often criticised as “black box” algorithms, generating predictions that cannot be explained due to their complex internal workings. Our implementation mitigates this by generating heatmaps, highlighting the CT image pixels that contribute most significantly to the final model predictions. This can aid users in identifying whether the model is placing undue importance on insignificant image features—the ability to identify errors and fallacies is a step towards producing a correctable and dependable system. Additionally, this implementation enables visual localisation of pathology, without the need for obtaining radiologist-labelled pixel-level annotations of haemorrhagic areas for the model to train on.

Our model performed with high accuracy, achieving AUC-ROCs greater than or equal to 0.949 on all classes. EDH, SAH and SDH were the poorest performing classes based on both AUC-ROC and AUC-PR. Notably, the ground truth radiologist labelling of these three subtypes also had the lowest inter-rater reliability (Fleiss *κ* 0.61, 0.64, and 0.54, respectively). Given the subjectivity of visual human interpretation, these ground truth labels are not free of error. Hence, the effects of potentially erroneous ground truth labels on DL model evaluation should be considered.

This study had several limitations. Firstly, the influence of haemorrhage mimics, including intracranial calcifications, cavernous haemangiomas, acute clots, and post-treated lesions such as embolised arteriovenous malformations and tumours, had not been evaluated in this study. These mimics may have played a role in reducing model accuracy. Secondly, the model performance had not been tested on images subject to different CT image reconstruction methods. Iterative reconstruction techniques have been used to improve image noise and image quality over traditional filtered back projection methods, and these techniques are known to result in perceptible differences in images presented to the reader [33, 34]. Hence, future work to investigate the impacts of these techniques on model performance may be worthwhile. Thirdly, the datasets used contained class imbalances, with disproportionately fewer scans containing EDH compared to the other haemorrhage subtypes in both Kaggle (354, 1.6%) and CQ500 (13, 2.6%) datasets. During testing, this led to wide confidence intervals for the sensitivity, AUC-ROC, and AUC-PR for EDH detection. Notably, EDH can also be more challenging to detect due to proximity to the adjacent hyperdense calvarium. This problem is compounded with the use of thicker image slices, which suffer from volume averaging artefacts [35]. Given that our DL model had ten times fewer EDH-containing images to train on, compared to the other subtypes, our model performance on EDH detection could potentially be improved by acquiring more images.

The prevalence of intracranial haemorrhage in both datasets (almost 60%) was not reflective of real-life clinical populations. ICH has an overall incidence of 24.6 per 100,000 person-years [1] with other subtypes such as SAH being less common [25, 36]. Although sensitivity, specificity and AUC-ROC are independent of prevalence, precision and AUC-PR are sensitive to it, as demonstrated by the significantly lower AUC-PR for EDH (0.584) compared to its AUC-ROC (0.971). AUC-PR has been argued to be a better evaluation metric than AUC-ROC in imbalanced datasets such as these where certain haemorrhage subtypes are more common, [23–25]; however, the ability to generalise these specific metrics to realistic clinical populations is limited. Hence, likelihood ratios were also computed. Given a pre-test probability (*i.e.,* prevalence) of haemorrhage, the likelihood ratio can be used to compute the post-test probability of haemorrhage in the case of a positive prediction. However, the prevalence/rates of haemorrhage (and its subtypes) amongst CT head scans performed in a hospital is currently unclear in the literature. Hence, a study clarifying these prevalences, or a direct evaluation of the model in a clinical setting—reflective of the true target population—is required.

Aside from this, future directions for this DL system relate to its clinical utility. Clinical deployment necessitates integration with clinical workflow tools such as radiology information and picture archiving and communication systems (RIS-PACS). Our model has been shown to rapidly generate predictions; however, this does not take into account the additional time involved with reciprocal data transfer from the DL device to the clinical RIS-PACS tools. Additionally, following integration, the ease of use of the system by practising physicians must also be evaluated. Furthermore, our DL model is currently limited to the detection, subtyping and localisation of intracranial haemorrhage. However, quantification of intracranial haemorrhage volume is also important for estimating the burden of disease and weighing management options [37, 38]. Thus, it would be worthwhile to incorporate such a feature into future DL models.

To summarise, this study demonstrates the high performance of a DL model for the automatic detection, subtyping and localisation of intracranial haemorrhage on NCCT head studies. The use of multiple image preprocessing techniques substantially improved the performance of the model, highlighting the need for greater emphasis on understanding the quality of data that is fed into DL models. Furthermore, the addition of a technique to visualise the model predictions provides an opportunity to explain and rationalise its predictions. The implementation of this model into a triage role has the potential to improve the diagnostic yield and efficiency of CT reporting, thus expediting treatment and improving outcomes for intracranial haemorrhage. Further testing of the model on prospective data, while it is integrated with clinical workflow systems, will be integral to evaluate its clinical utility.

## Supplementary Information

**Additional file 1:** **Supplementary Table 1.** Inter-rater reliability of radiologist labels for the CQ500 dataset. **Supplementary Table 2.** Scan acquisition information and collection methods for the Kaggle and CQ500 datasets. **Supplementary Table 3.** Performances of each trained model on each haemorrhage class. **Supplementary Table 4.** *p*-values calculated from DeLong’s test and McNemar’s test for comparison between models on detection of any intracranial haemorrhage. **Supplementary Figure 1.** Examples of image preprocessing using image windowing. Demonstration of the preprocessing pipeline using the image windowing technique, on two example inputs A and B (top row and bottom row, respectively). Each DICOM CT slice was set to a specific window setting: brain window (WL = 40, WW = 80) (first column), soft tissue window (WL = 40, WW = 380) (second column), and subdural window (WL = 80, WW = 200) (third column). The final preprocessed image (fourth column) contains these three windowed images, where each channel of the output three-channel 8-bit JPEG image corresponds to each windowed image. **Supplementary Figure 2.** Examples of image preprocessing using slice concatenation. Demonstration of the preprocessing pipeline using the slice concatenation technique, on two example inputs A and B (top row and bottom row, respectively). For each DICOM CT slice, the slice immediately superior (third column) and the slice immediately inferior (first column) to the current slice (second column) were obtained. These slices were set to the brain window setting. The final preprocessed image (fourth column) contains these three slice images, where each channel of the output three-channel 8-bit JPEG image corresponds to each slice. **Supplementary Figure 3.** Illustration of the CNN-RNN architecture used. From each CT study, each input image slice was analysed by the CNN. The CNN was composed of a ResNeXt-101 backbone. The CNN’s outputs (obtained from the final global average pooling layer, immediately before the final fully connected layer), were used as input into the RNN. The RNN was composed of two stacked bi-directional LSTM layers, each with 2048 features in the hidden state. Linear layers were also used. The LSTM and linear layers were summed together, before being passed through a final linear layer to convert the output vectors into logits for each class of haemorrhage (study-level prediction).

## Data Availability

As open-source data was used, they are available for download online (Kaggle dataset: https://www.kaggle.com/competitions/rsna-intracranial-haemorrhage-detection/data, CQ500 dataset: http://headctstudy.qure.ai/dataset). The code used is available on GitHub (https://github.com/melissa-yeo/CT-haemorrhage-classification).

## Abbreviations

2D: Two-dimensional
3D: Three-dimensional
AUC-PR: Area under the precision-recall curve
AUC-ROC: Area under the receiver operating characteristic curve
CNN: Convolutional neural network
CNN_ens_: Ensemble of CNN_slc_ and CNN_wdw_
CNN_slc_: CNN trained on slice-concatenated preprocessed images
CNN_wdw_: CNN trained on windowed preprocessed images
CT: Computed tomography
DL: Deep learning
EDH: Extradural haemorrhage
Grad-CAM: Gradient-weighted class activation mapping
ICH: Intracerebral haemorrhage
IVH: Intraventricular haemorrhage
mAP: Microaveraged precision score
NCCT: Non-contrast computed tomography
PR: Precision-recall
RIS-PACS: Radiology information and picture archiving and communication systems
RNN: Recurrent neural network
ROC: Receiver operating characteristic
SAH: Subarachnoid haemorrhage
SDH: Subdural haemorrhage
WL: Window level
WW: Window width
