# Supplementary material for: Evaluation of techniques to improve a deep learning algorithm for the automatic detection of intracranial haemorrhage on CT head imaging
Source: Eur Radiol Exp. 2023 Apr 10;7:17. doi: 10.1186/s41747-023-00330-3 (PMC10083149; doi:10.1186/s41747-023-00330-3)

ELECTRONIC SUPPLEMENTARY MATERIAL

Evaluation of techniques to improve a deep learning algorithm for the automatic detection of intracranial haemorrhage on CT head imaging

Supplementary Table 1 – Inter-rater reliability of radiologist labels for the CQ500 dataset

	**R1 and R2**		**R1 and R3**		**R2 and R3**		**All raters (R1-R3)**
	Agreement (n, %)	Cohen's kappa coefficient (κ)	Agreement (n, %)	Cohen's kappa coefficient (κ)	Agreement (n, %)	Cohen's kappa coefficient (κ)	Fleiss' kappa coefficient
**Intracranial haemorrhage**	437 (89%)	0.78	434 (88%)	0.76	446 (91%)	0.81	0.78
**EDH**	478 (97%)	0.51	482 (98%)	0.60	483 (98%)	0.73	0.61
**ICH**	448 (91%)	0.79	446 (91%)	0.77	445 (91%)	0.77	0.78
**IVH**	472 (96%)	0.70	470 (96%)	0.66	477 (97%)	0.73	0.70
**SAH**	457 (93%)	0.68	446 (91%)	0.64	446 (91%)	0.61	0.64
**SDH**	432 (88%)	0.49	442 (90%)	0.56	457 (93%)	0.60	0.54

Each radiologist/ rater is referred to as R1, R2 and R3 respectively.
Agreement is in n scans (%). Cohen's kappa coefficient and Fleiss' Kappa coefficient: 0.41-0.60 indicates moderate agreement, 0.61-0.80 indicates substantial agreement, 0.81-0.99 indicates near perfect agreement.
Abbreviations: EDH = extradural haemorrhage, ICH = intracerebral haemorrhage, IVH = intraventricular haemorrhage, SAH = subarachnoid haemorrhage, SDH = subdural haemorrhage.

Supplementary Table 2 – Scan acquisition information and collection methods for the Kaggle and CQ500 datasets

	Training (Kaggle dataset)	Test (CQ500 dataset)
**Number of volumetric studies**	21,744	491
**Number of 2D slice images**	752,803	193,317
**Median slice thickness in mm (range)**	5 (1 – 7)	5 (0.625 – 5)
**Median number of slices per study (range)**	34 (20 – 548)	32 (12 – 413)
**2D slice image resolution (pixel)**	512 x 512	512 x 512
**Collection centres/ institutions**	▪ Stanford University Center for Artificial Intelligence in Medicine & Imaging (AIMI)<br>▪ St. Michael's Hospital Li Ka Shing Centre for Healthcare Analytics Research & Training (LKS-CHART)<br>▪ Thomas Jefferson University Department of Radiology<br>▪ Universidade Federal de São Paulo (Unifesp)	▪ Six radiology centres in New Delhi (outpatient and hospital radiology departments)
**Collection period**	Unknown	1 Jan 2012 – 1 Feb 2018
**CT scanner models**	Unknown	GE BrightSpeed, GE Discovery CT750 HD, GE LightSpeed, GE Optima CT660, Philips MX 16-slice, Philips Access-32 CT

Supplementary Table 3 – Performances of each trained model on each haemorrhage class

		AUC-PR	AUC-ROC
**Intracranial haemorrhage**	CNN	0.868 (0.825-0.903)	0.854 (0.816-0.889)
	CNN (wdw)	0.959 (0.941-0.977)	0.964 (0.948-0.979)
	CNN (slc)	0.964 (0.947-0.979)	0.967 (0.952-0.980)
	CNN (ens)	0.964 (0.947-0.979)	0.967 (0.953-0.981)
	CNN-RNN	0.867 (0.819-0.912)	0.871 (0.835-0.903)
	CNN-RNN (wdw)	0.961 (0.942-0.977)	0.963 (0.947-0.977)
	CNN-RNN (slc)	0.962 (0.944-0.978)	0.967 (0.952-0.979)
	CNN-RNN (ens)	0.965 (0.948-0.979)	0.966 (0.951-0.980)
**EDH**	CNN	0.427 (0.038-0.841)	0.875 (0.702-0.995)
	CNN (wdw)	0.481 (0.097-0.878)	0.971 (0.931-0.998)
	CNN (slc)	0.481 (0.039-0.948)	0.936 (0.856-0.999)
	CNN (ens)	0.505 (0.065-0.948)	0.963 (0.918-0.999)
	CNN-RNN	0.374 (0.019-0.804)	0.819 (0.621-0.985)
	CNN-RNN (wdw)	0.590 (0.137-0.950)	0.981 (0.952-0.999)
	CNN-RNN (slc)	0.571 (0.100-1.000)	0.975 (0.933-1.000)
	CNN-RNN (ens)	0.584 (0.117-1.000)	0.971 (0.931-1.000)
**ICH**	CNN	0.868 (0.801-0.924)	0.937 (0.910-0.962)
	CNN (wdw)	0.945 (0.914-0.971)	0.982 (0.973-0.990)
	CNN (slc)	0.930 (0.895-0.960)	0.970 (0.957-0.983)
	CNN (ens)	0.943 (0.911-0.969)	0.980 (0.970-0.989)
	CNN-RNN	0.865 (0.799-0.928)	0.940 (0.915-0.964)
	CNN-RNN (wdw)	0.950 (0.915-0.975)	0.983 (0.973-0.991)
	CNN-RNN (slc)	0.941 (0.908-0.968)	0.978 (0.967-0.988)
	CNN-RNN (ens)	0.951 (0.919-0.974)	0.983 (0.973-0.990)
**IVH**	CNN	0.883 (0.777-0.960)	0.967 (0.915-0.996)
	CNN (wdw)	0.926 (0.848-0.977)	0.989 (0.975-0.998)
	CNN (slc)	0.908 (0.827-0.969)	0.982 (0.963-0.996)
	CNN (ens)	0.923 (0.844-0.976)	0.990 (0.979-0.997)
	CNN-RNN	0.925 (0.852-0.979)	0.973 (0.928-0.998)
	CNN-RNN (wdw)	0.942 (0.883-0.982)	0.992 (0.983-0.998)
	CNN-RNN (slc)	0.924 (0.849-0.976)	0.984 (0.966-0.997)
	CNN-RNN (ens)	0.934 (0.871-0.979)	0.991 (0.980-0.998)
**SAH**	CNN	0.665 (0.588-0.738)	0.787 (0.735-0.839)
	CNN (wdw)	0.872 (0.824-0.916)	0.926 (0.896-0.955)
	CNN (slc)	0.899 (0.856-0.936)	0.944 (0.920-0.966)
	CNN (ens)	0.897 (0.853-0.935)	0.940 (0.913-0.965)
	CNN-RNN	0.677 (0.590-0.757)	0.811 (0.761-0.859)
	CNN-RNN (wdw)	0.883 (0.837-0.926)	0.948 (0.925-0.967)
	CNN-RNN (slc)	0.900 (0.858-0.936)	0.952 (0.932-0.969)
	CNN-RNN (ens)	0.889 (0.844-0.930)	0.949 (0.927-0.967)
**SDH**	CNN	0.688 (0.596-0.771)	0.814 (0.754-0.869)
	CNN (wdw)	0.867 (0.801-0.924)	0.948 (0.915-0.976)
	CNN (slc)	0.859 (0.789-0.918)	0.944 (0.909-0.971)
	CNN (ens)	0.867 (0.797-0.926)	0.948 (0.917-0.976)

	CNN-RNN	0.724 (0.635-0.804)	0.835 (0.777-0.891)
	CNN-RNN (wdw)	0.881 (0.818-0.931)	0.948 (0.916-0.976)
	CNN-RNN (slc)	0.876 (0.812-0.928)	0.951 (0.922-0.976)
	CNN-RNN (ens)	0.892 (0.830-0.943)	0.953 (0.922-0.979)

95% CIs are provided in parentheses. CNN denotes models composed of purely a CNN. CNN-RNN denotes models composed of a joint CNN-RNN. (wdw) denotes models trained with preprocessed windowed images. (slc) denotes models trained with preprocessed slice concatenated images. (ens) denotes ensemble models created by combining the model trained with preprocessed windowed images and the model trained with preprocessed slice concatenated images.

Abbreviations: CNN = convolutional neural network, DL = deep learning, EDH = extradural haemorrhage, ICH = intracerebral haemorrhage, IVH = intraventricular haemorrhage, RNN = recurrent neural network, SAH = subarachnoid haemorrhage, SDH = subdural haemorrhage.

Supplementary Table 4 – P-values calculated from DeLong's test and McNemar's test for comparison between models on detection of any intracranial haemorrhage

	Model comparison	p-value (DeLong's test)	p-value (McNemar's test)
Comparing effect of windowed input images	CNN vs CNN (wdw)	5.10×10^{-12}	2.47×10^{-29}
	CNN-RNN vs CNN-RNN (wdw)	3.27×10^{-10}	1.70×10^{-12}
	CNN (slc) vs CNN (ens)	$\mathbf{8.23 \times 10^{-1}}$	$\mathbf{5.22 \times 10^{-2}}$
	CNN-RNN (slc) vs CNN-RNN (ens)	$\mathbf{9.70 \times 10^{-1}}$	$\mathbf{1.34 \times 10^{-1}}$
Comparing effect of slice concatenated input images	CNN vs CNN (slc)	1.90×10^{-12}	8.15×10^{-37}
	CNN-RNN vs CNN-RNN (slc)	3.38×10^{-11}	1.05×10^{-8}
	CNN (wdw) vs CNN (ens)	$\mathbf{6.48 \times 10^{-2}}$	1.35×10^{-2}
	CNN-RNN (wdw) vs CNN-RNN (ens)	$\mathbf{1.40 \times 10^{-1}}$	$\mathbf{7.68 \times 10^{-2}}$
Comparing effect of additional RNN	CNN vs CNN-RNN	1.68×10^{-2}	**1.00**
	CNN (wdw) vs CNN-RNN (wdw)	$\mathbf{9.22 \times 10^{-1}}$	1.39×10^{-17}
	CNN (slc) vs CNN-RNN (slc)	$\mathbf{9.02 \times 10^{-1}}$	5.23×10^{-30}
	CNN (ens) vs CNN-RNN (ens)	$\mathbf{7.50 \times 10^{-1}}$	2.25×10^{-24}
Comparing effect of all preprocessing with addition of RNN	CNN vs CNN-RNN (ens)	3.91×10^{-12}	1.28×10^{-9}

Numbers in bold indicate failure to reject the null hypothesis. CNN denotes models composed of purely a CNN. CNN-RNN denotes models composed of a joint CNN-RNN. (wdw) denotes models trained with preprocessed windowed images. (slc) denotes models trained with preprocessed slice concatenated images. (ens) denotes ensemble models created by combining the model trained with preprocessed windowed images and the model trained with preprocessed slice concatenated images.

Abbreviations: CNN = convolutional neural network, RNN = recurrent neural network.

Supplementary Figure 1 – Examples of image preprocessing using image windowing

Demonstration of the preprocessing pipeline using the image windowing technique, on two example inputs A and B (top row and bottom row, respectively). Each DICOM CT slice was set to a specific window setting: brain window (WL = 40, WW = 80) (first column), soft tissue window (WL = 40, WW = 380) (second column), and subdural window (WL = 80, WW = 200) (third column). The final preprocessed image (fourth column) contains these three windowed images, where each channel of the output three-channel 8-bit JPEG image corresponds to each windowed image.

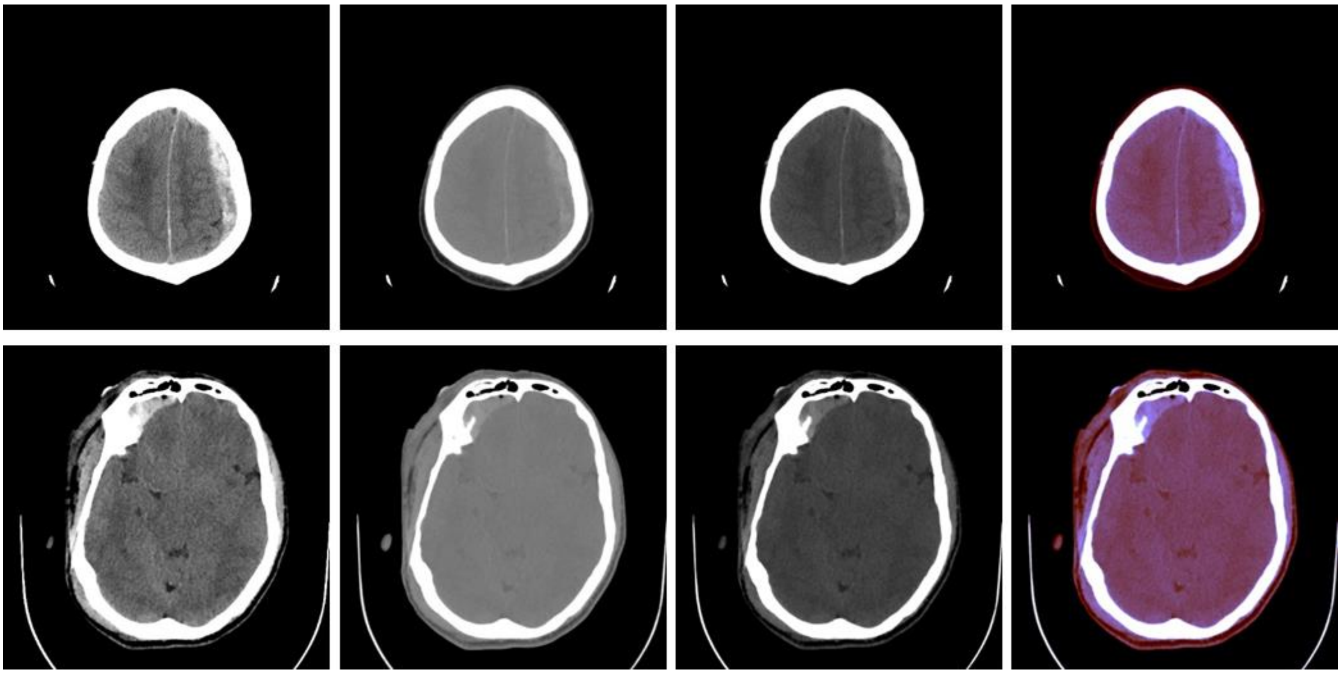

Supplementary Figure 2 – Examples of image preprocessing using slice concatenation

Demonstration of the preprocessing pipeline using the slice concatenation technique, on two example inputs A and B (top row and bottom row, respectively). For each DICOM CT slice, the slice immediately superior (third column) and the slice immediately inferior (first column) to the current slice (second column) were obtained. These slices were set to the brain window setting. The final preprocessed image (fourth column) contains these three slice images, where each channel of the output three-channel 8-bit JPEG image corresponds to each slice.

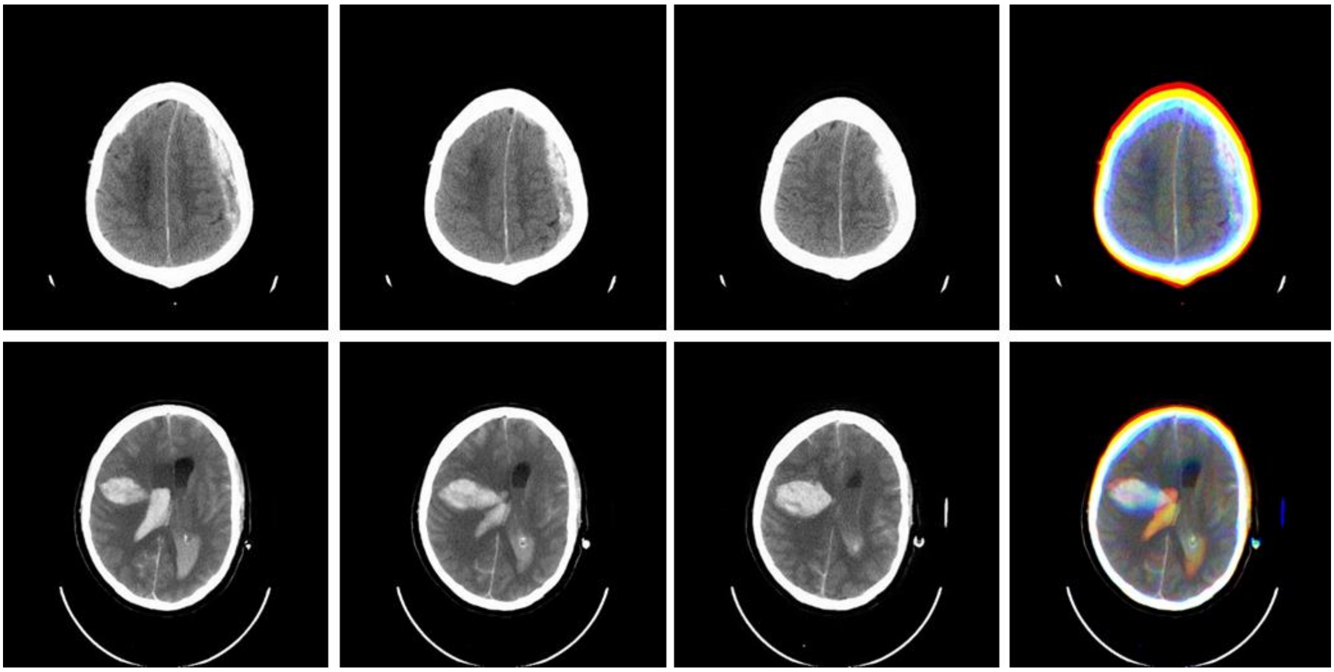

Supplementary Figure 3 – Illustration of the CNN-RNN architecture used

From each CT study, each input image slice was analysed by the CNN. The CNN was composed of a ResNeXt-101 backbone. The CNN's outputs (obtained from the final global average pooling layer, immediately before the final fully connected layer), were used as input into the RNN. The RNN was composed of two stacked bi-directional LSTM layers, each with 2048 features in the hidden state. Linear layers were also used. The LSTM and linear layers were summed together, before being passed through a final linear layer to convert the output vectors into logits for each class of haemorrhage (study-level prediction).

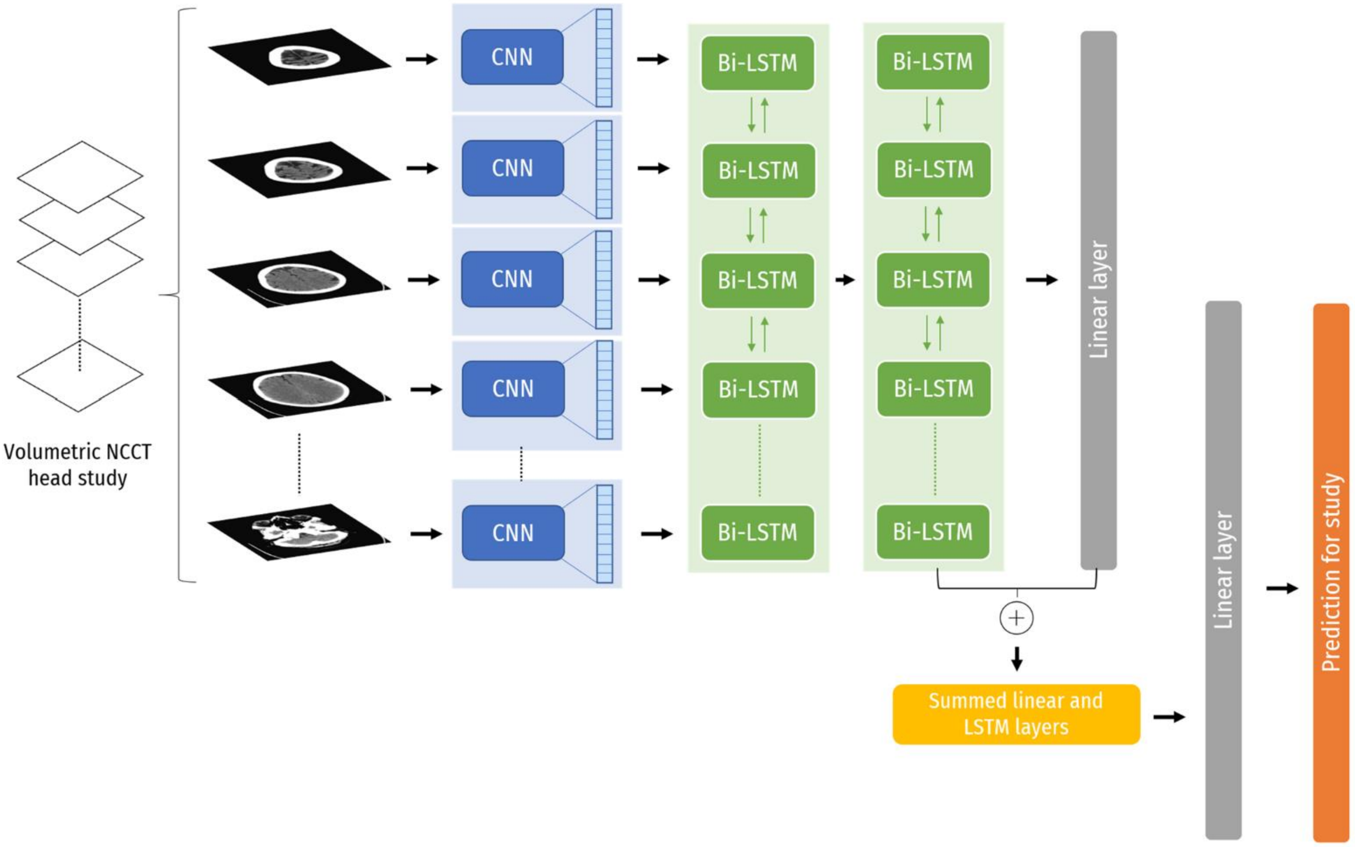

Supplement: Supplementary file 1 — Additional file 1: Supplementary Table 1. Inter-rater reliability of radiologist labels for the CQ500 dataset. Supplementary Table 2. Scan acquisition information and collection methods for the Kaggle and CQ500 datasets. Supplementary Table 3. Performances of each trained model on each haemorrhage class. Supplementary Table 4. p-values calculated from DeLong’s test and McNemar’s test for comparison between models on detection of any intracranial haemorrhage. Supplementary Figure 1. Examples of image preprocessing using image windowing. Demonstration of the preprocessing pipeline using the image windowing technique, on two example inputs A and B (top row and bottom row, respectively). Each DICOM CT slice was set to a specific window setting: brain window (WL = 40, WW = 80) (first column), soft tissue window (WL = 40, WW = 380) (second column), and subdural window (WL = 80, WW = 200) (third column). The final preprocessed image (fourth column) contains these three windowed images, where each channel of the output three-channel 8-bit JPEG image corresponds to each windowed image. Supplementary Figure 2. Examples of image preprocessing using slice concatenation. Demonstration of the preprocessing pipeline using the slice concatenation technique, on two example inputs A and B (top row and bottom row, respectively). For each DICOM CT slice, the slice immediately superior (third column) and the slice immediately inferior (first column) to the current slice (second column) were obtained. These slices were set to the brain window setting. The final preprocessed image (fourth column) contains these three slice images, where each channel of the output three-channel 8-bit JPEG image corresponds to each slice. Supplementary Figure 3. Illustration of the CNN-RNN architecture used. From each CT study, each input image slice was analysed by the CNN. The CNN was composed of a ResNeXt-101 backbone. The CNN’s outputs (obtained from the final global average pooling layer, immediately b [file 41747_2023_330_MOESM1_ESM.pdf]
